# Proteomic Discovery of VEEV E2-Host Partner Interactions Identifies GRP78 Inhibitor HA15 as a Potential Therapeutic for Alphavirus Infections

**DOI:** 10.3390/pathogens10030283

**Published:** 2021-03-02

**Authors:** Michael D. Barrera, Victoria Callahan, Ivan Akhrymuk, Nishank Bhalla, Weidong Zhou, Catherine Campbell, Aarthi Narayanan, Kylene Kehn-Hall

**Affiliations:** 1 National Center for Biodefense and Infectious Diseases, School of Systems Biology, George Mason University, Manassas, VA 20110, USA; mbarrer@masonlive.gmu.edu (M.D.B.); vcallah@gmu.edu (V.C.); nbhalla@gmu.edu (N.B.); anaraya1@gmu.edu (A.N.); 2 Department of Biomedical Sciences and Pathobiology, Virginia Polytechnic Institute and State University, Blacksburg, VA 24061, USA; Iakhrymu@vt.edu; 3 Center for Applied Proteomics and Molecular Medicine, School of Systems Biology, George Mason University, Manassas, VA 20110, USA; wzhou@gmu.edu; 4 DCE Consulting, Vienna, VA 22181, USA; ccgables@verizon.net

**Keywords:** E2, glycoprotein, GRP78, alphavirus, therapeutic, proteomics

## Abstract

Alphaviruses are a genus of the *Togaviridae* family and are widely distributed across the globe. Venezuelan equine encephalitis virus (VEEV) and eastern equine encephalitis virus (EEEV), cause encephalitis and neurological sequelae while chikungunya virus (CHIKV) and Sindbis virus (SINV) cause arthralgia. There are currently no approved therapeutics or vaccines available for alphaviruses. In order to identify novel therapeutics, a V5 epitope tag was inserted into the N-terminus of the VEEV E2 glycoprotein and used to identify host-viral protein interactions. Host proteins involved in protein folding, metabolism/ATP production, translation, cytoskeleton, complement, vesicle transport and ubiquitination were identified as VEEV E2 interactors. Multiple inhibitors targeting these host proteins were tested to determine their effect on VEEV replication. The compound HA15, a GRP78 inhibitor, was found to be an effective inhibitor of VEEV, EEEV, CHIKV, and SINV. VEEV E2 interaction with GRP78 was confirmed through coimmunoprecipitation and colocalization experiments. Mechanism of action studies found that HA15 does not affect viral RNA replication but instead affects late stages of the viral life cycle, which is consistent with GRP78 promoting viral assembly or viral protein trafficking.

## 1. Introduction

Alphaviruses are a genus of the *Togaviridae* family that are significant human and veterinary pathogens. Venezuelan equine encephalitis virus (VEEV) and eastern equine encephalitis virus (EEEV) are endemic to the Western Hemisphere. More specifically, VEEV is endemic to the United States, Central and South America [1], while EEEV is endemic to North, Central, and South America and the Caribbean [2]. Sindbis virus (SINV) is widely distributed and found in Africa, Asia, Europe, and Australia while chikungunya virus (CHIKV) is endemic to Africa, South American and Central America [3,4]. In humans, alphaviruses cause general symptoms of malaise, fever, and headaches, but alphaviruses, such as VEEV and EEEV are more neurovirulent and can cause encephalitis and neurological sequelae whereas SINV and CHIKV cause arthralgia [3,5,6].

VEEV and EEEV pose a threat through their reemergence and potential use as biological weapons. As recently as 2019 an outbreak of EEEV occurred in the United States where 36 human cases were reported and 14 of those cases were fatal [7]. VEEV and EEEV can be grown to high titers, cause infection by aerosolization, and have significant incidence of morbidity and mortality, especially with respect to EEEV. These characteristics, along with the lack of vaccines and therapeutics, result in them being classified as Category B priority pathogens by the National Institute of Allergy and Infectious Diseases (NIAID) and select agents by both the Centers for Disease Control (CDC) and the United States Department of Agriculture (USDA).

Alphaviruses are enveloped viruses with a positive-sense single stranded RNA genome of about 11.5 kb. The first open reading frame (ORF) found on the genomic RNA encodes the four non-structural proteins nsP1-4. The 26S subgenomic promoter found on the negative strand of the genome controls the production of the 26S subgenomic RNA. The 26S subgenomic RNA contains the second ORF which encodes the structural proteins capsid, E3, E2, 6K/TF, and E1.

E1/E2 glycoproteins comprise the viral spike protein, which consists of trimers of E1/E2 heterodimer spikes [8]. E2 is responsible for receptor binding which leads to clathrin mediated endocytosis. After acidification of the endosome, E2 and E1 dissociate allowing E1 to fuse the viral envelope with the endosomal membrane and release the nucleocapsid. nsP1-4 are immediately translated from the incoming viral RNA and are responsible for the replication of the viral RNA and production of the 26S subgenomic RNA. After the structural polyprotein is translated from the 26S subgenomic RNA, the capsid’s autoprotease activity cleaves it from the growing polyprotein and reveals the ER localization sequence on E3. The remaining structural proteins are translated on the ER and are trafficked through the Golgi and to the plasma membrane. During this process E2 undergoes cleavage from E3 and after folding E2 and E1 form a heterodimer. At the plasma membrane E2 proteins interact with the nucleocapsid and the energy from that interaction allows for the release of the virion [3,9].

The development of new therapeutics against alphaviruses is critical due to the reemergence of these viruses and their potential use as biological weapons. Targeting host proteins is an attractive therapeutic option as these inhibitors tend to have broad-spectrum activity and result in the development of less viral resistance as compared to direct-acting antivirals. There are a number of E2—host protein interactions that have been elucidated to date [reviewed in [10]]. E2 can interact with Dendritic Cell-Specific Intercellular adhesion molecule-3-Grabbing Non-integrin (DC-SIGN), liver/lymph node-specific intracellular adhesion molecules-3 grabbing non-integrin (L-SIGN) or Heparin sulfate to facilitate viral entry [11,12,13]. VEEV E2 colocalizes with Ras-related C3 botulinum toxin substrate 1 (Rac1) and Phosphatidylinositol-4-phosphate 5-kinase type-1 (PIP5K1-α) and uses the host proteins actin, actin related protein 3 (Arp3), and Rac1 to enable its trafficking to the plasma membrane [14].

Given the critical role of the E2 protein for viral entry and budding, it is anticipated that there are additional host-protein interactions yet to be discovered. In this study, a V5 tag attached to the N-terminus of the VEEV E2 glycoprotein was used to enable proteomic identification of VEEV E2-host protein interactions. Various inhibitors of the identified host protein interactors were tested and the compound HA15, an inhibitor of the ER chaperone GRP78 developed by Cerezo et al. 2016 [15], was found to be the most effective. HA15 reduced VEEV infectious titers and displayed broad-spectrum alphaviruses activity against EEEV, SINV, and CHIKV. GRP78 and VEEV E2 were confirmed as interacting partners through co-localization and co-immunoprecipitations assays. Inhibition of GRP78 resulted in reduced production of VEEV through targeting a late stage of the viral life cycle as evidenced by decreased infectious titers, but no significant inhibition of viral RNA levels.

## 2. Results

### 2.1. Characterization of VEEV TC-83 Expressing E2 with a N-Terminal V5-Epitope Tag

**Figure 1:**
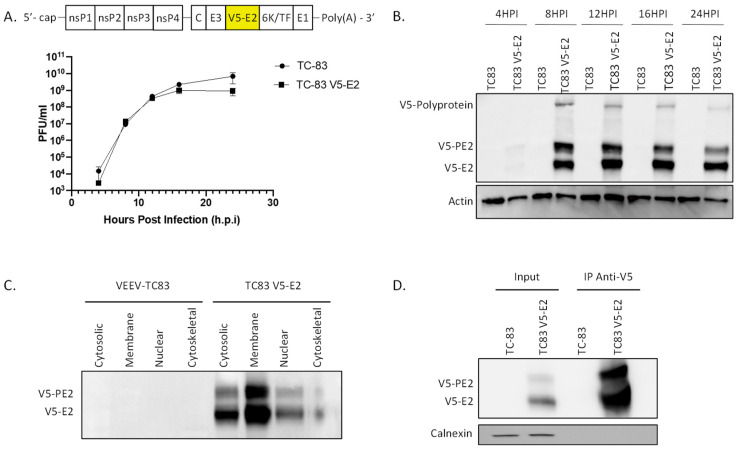
Characterization of VEEV TC-83 expressing E2 with a N-terminal V5-epitope tag. (**A**) Schematic of VEEV TC-83 with V5 tag inserted into the N terminus of the E2 glycoprotein and replication kinetics for the parental VEEV TC-83 virus and the VEEV TC-83 V5-E2 virus in HEK293T cells. Values are an average of 3 biological replicates ± standard deviation. (**B**) HEK293T cells were infected with VEEV TC-83 or VEEV TC-83 V5-E2 and whole cell lysates were collected at 4, 8, 12, 16, and 24 hpi. Lysates were analyzed by western blot and probed for V5 and actin as a loading control. (**C**) HEK293T cells were infected with VEEV TC-83 or VEEV TC-83 V5-E2 at a MOI of 5. Cells were collected at 12 hpi and fractionated with a Qiagen QProteome Cell Compartment kit and analyzed using western blot and probed for V5. (**D**) The membrane fractions from panel C was immunoprecipitated using an anti-V5 antibody and the precipitate was analyzed by western blot probed for V5 and calnexin.

To facilitate protein interaction analysis, a V5 epitope tag was added to the N terminus of the VEEV E2 glycoprotein in the context of the VEEV TC-83 genome, referred to as VEEV TC-83 V5-E2 (Figure 1A). To determine the effects of the inserted tag on viral replication kinetics and E2 expression, the virus was grown in HEK 293T cells. The viral growth kinetics of VEEV TC-83 V5-E2 were not significantly different from the parental VEEV-TC83 (Figure 1A). After 12 h post infection (hpi) both viruses reached a titer of over 1 × 10^8^ plaque forming units (PFU)/mL and both plateaued at 24 hpi attaining titers ≥ 1 × 10^9^ PFU/mL. Cell lysates from HEK 293T cells infected with VEEV TC-83 and TC-83 V5-E2 were analyzed by western blot analysis (Figure 1B). Robust E2 expression was observed starting at 8 hpi. Three bands are present in the lanes containing lysates from cells infected with VEEV TC-83 V5-E2, the lower band corresponds to the E2 protein, the middle band to the PE2 protein (polyprotein of E3 and E2), and the top band corresponds to the polyprotein. No protein resembling the V5 sequence was found in lysates from cells infected with the parental VEEV-TC83. The data shows that the V5 tag does not affect the viral kinetics of VEEV-TC83 within the cell.

### 2.2. VEEV E2 Interactome Characterization

To determine protein interactions between VEEV E2 and the host cell proteins, VEEV TC-83 V5-E2 was used to infect HEK 293T cell. The cells were infected at a MOI of 5 and the cell lysates were collected at 12 hpi. Lysates were fractionated using the Qiagen QProteome Cell Compartment kit to enrich for cell fractions containing E2 protein complexes. As expected, the membrane fraction contained the highest concentration of V5-E2 protein (Figure 1C) and was used for the immunoprecipitation of the V5-E2 protein (Figure 1D) and interacting host proteins. Immunoprecipitated samples were subjected to LC-MS/MS to identify E2 interacting host partners. VEEV TC-83 infected lysates and mock infected cells incubated with anti-V5 antibody were analyzed in parallel as negative controls. Proteins identified through LC-MS/MS in the negative control samples were subtracted from the proteins identified in the immunoprecipitated samples from VEEV TC-83 V5-E2 infected cells (experimental samples). This analysis resulted in the identification of 785 E2 interacting proteins, which includes isoforms (Table S1). LC-MS/MS data were imported into Ingenuity Pathway Analysis (IPA) software, which consolidates the isoforms, resulting in 155 interacting proteins remaining. Finally, this number was further refined to only include protein interactions which were present in 3 out of 3 biological repeats, resulting in 21 protein interactors. The 21 interacting proteins that appear in all three replicates are shown in Table 1. These proteins can be further categorized as being involved in protein folding, metabolism/ATP production, translation, cytoskeleton, complement, vesicle transport and ubiquitination. From those results the interaction with the 78kDa glucose-regulated protein (GRP78), heat shock 70 kDa protein (HSP70), ATP synthase, and Ras-related protein Rab-1 were chosen for further study. These proteins were chosen based on inhibitors being readily available to assess their potential importance in VEEV replication.

### 2.3. Inhibition of GRP78 through HA15 Treatment Reduces VEEV TC-83 Infectious Titers

**Figure 2:**
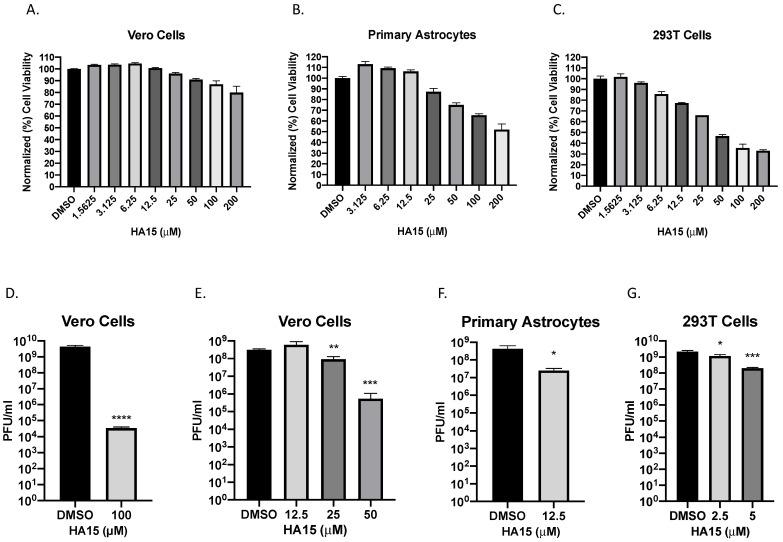
Inhibition of GRP78 through HA15 treatment reduces VEEV TC-83 infectious titers. (**A**–**C**) Cytotoxicity of HA15 was determined in Vero cells, primary human astrocytes, and HEK293T cells using a CellTiter-Glo Luminescent Cell Viability Assay after 24 h of treatment with the inhibitors. (**D**) Effects of the inhibitors on infectious viral titers in Vero cells. Cells were pretreated for one hour with the inhibitor and then infected with VEEV TC-83 (MOI of 0.1) for one hour. After infection the inhibitor was reapplied and the supernatant was collected at 16 hpi. Viral infectivity was measured by plaque assay. (**E**) Dose-dependent effects of HA15 was determined in Vero cells following the procedures described in (**D**). (**F**,**G**) Effects of HA15 on VEEV TC-83 titers of human primary astrocytes and HEK293T cells. Cells were treated and infected as described in (**D**). Values are an average of 3 biological replicates ± standard deviation. * *p* < 0.05, ** *p* < 0.01, *** *p* < 0.001, **** *p* < 0.0001.

To determine the importance of the identified E2-host protein interactions, a total of six inhibitors were tested, Fluvastatin (Rab-1 inhibitor), VER155008 (HSP70 inhibitor), BTB06584 (ATP synthase inhibitor), Enterostatin (ATP synthase inhibitor), Metformin (GRP78 inhibitor), and HA15 (GRP78 inhibitor). Fluvastatin, VER155008, BTB06584, and Enterostatin had no impact on VEEV infectious titers using non-toxic concentrations in Vero cells (Figure S1). These data suggest that Rab-1, HSP70 and ATP synthase have limited impact on VEEV infectious titers. In contrast, inhibition of GRP78 through HA15 treatment, but not Metformin treatment significantly impacted VEEV infectious titers (Figure 2 and Figure S1). HA15 and Metformin had minimal impact on Vero cell viability with treatment of 100 µM of HA15 and Metformin resulting in cell viability of 87% and 99% respectively when normalized to the solvent control (Figure 2A and Figure S1A). Using non-toxic concentrations, the effects of these inhibitors on VEEV TC-83 titers were first assessed in Vero cells. Cells were pretreated with the inhibitors and subsequently infected with VEEV TC-83 at a MOI of 0.1 for one hour. Following infection, medium containing the inhibitors was added back to the cells. After 16 hpi viral titers were determined via plaque assay. Treatment of Vero cells with HA15 at 100 µM reduced VEEV TC-83 infectious titers by 5 log_10_ (Figure 2D). Metformin had no significant effect of VEEV TC-83 titers at 100 µM (Figure S1B). HA15 treatment of Vero cells inhibited VEEV TC-83 viral titers in a dose-dependent manner. Vero cells treated with 25 µM of HA15 resulted in viral titers being reduced by almost half a log from 3.12 × 10^8^ to 9.17 × 10^7^ PFU/mL, while 50 µM of HA15 resulted in a reduction of almost three logs, yielding 5.30 × 10^5^ PFU/mL (Figure 2E). The effects of HA15 in two human cell lines, primary human astrocytes and HEK 293T cells was also assessed. HA15 had a higher toxicity in these cell lines compared to the Vero cells (Figure 2B,C) and lower concentrations were selected for antiviral testing in these cell line. HA15 treatment in astrocytes and HEK 293T cells also significantly reduced the infectious titers of VEEV TC-83 (Figure 2F,G). In astrocytes, use of HA15 at a concentration of 12.5 µM decreased VEEV titers by ~1 log from 4.35 × 10^8^ to 2.53 × 10^7^ PFU/mL. In HEK 293T, HA15 treatment at 5 µM reduced VEEV titers by one log from 2.18 × 10^9^ to 2.02 × 10^8^ PFU/mL. These results show that inhibition of GRP78 by HA15 significantly impacted VEEV TC-83 infectious titers in multiple cell lines.

### 2.4. HA15 Is a Broad-Spectrum Alphavirus Inhibitor

**Figure 3:**
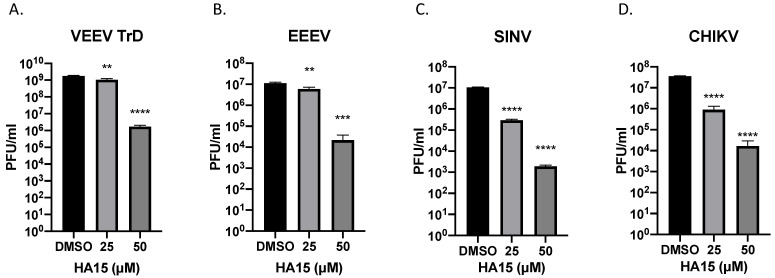
HA15 is a broad-spectrum alphavirus inhibitor. Vero cells were pretreated with DMSO control or HA15 (50 or 25 µM) for one hour and infected (**A**) VEEV TrD, (**B**) EEEV FL93-939, (**C**) SINV, or (**D**) CHIKV for one hour. Each infection was performed at a MOI of 0.1. Inhibitor treatments were reapplied after infection and supernatants collected at 16 hpi. Viral titers were determined by plaque assay. Values are an average of 3 biological replicates ± standard deviation. ** *p* < 0.01, *** *p* < 0.001, **** *p* < 0.0001.

To determine if GRP78 is important for other alphaviruses, the impact of HA15 on an additional strain of VEEV (VEEV TrD), EEEV FL93-939, SINV EgAr 339, and CHIKV 181/25 was assessed. For this analysis, Vero cells were pre- and post-treated with HA15 (50 µM and 25 µM) and infectious titers determined at 16 hpi. Both VEEV TrD and EEEV infectious viral titers were significantly impacted by HA15 treatment with a 3 log_10_ reduction observed in cells treated with 50 µM of HA15 (Figure 3A,B). HA15 treatment of SINV infected Vero cells at 50 µM and 25 µM reduced the viral titers from 2.18 × 10^7^ PFU/mL (DMSO treated cells) to 1.00 × 10^4^ PFU/mL and 1.43 × 10^6^ PFU/mL, respectively (Figure 3C). Likewise, HA15 treatment impacted CHIKV infectious viral titers, reducing them from 4.4 × 10^7^ PFU/mL (DMSO treated cells) to 3.33 × 10^4^ PFU/mL (HA15 50 µM) and 3.13 × 10^6^ PFU/mL (HA15 25µM) (Figure 3D). These results agree with the data demonstrating the ability of HA15 to inhibit VEEV TC-83 infectious titers (Figure 2E), indicating that HA15 inhibition of GRP78 impacts multiple alphaviruses.

### 2.5. E2 Glycoprotein of VEEV TC-83 Interacts with GRP78

**Figure 4:**
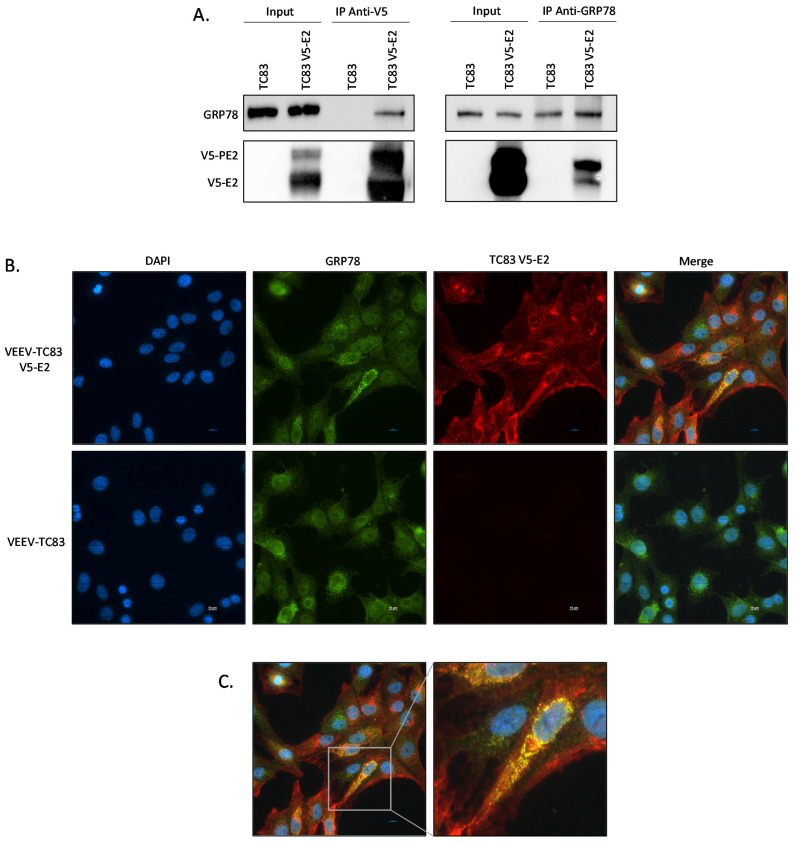
E2 Glycoprotein of VEEV TC-83 interacts with GRP78. (**A**) HEK293T cells were infected with VEEV TC-83 or V5 tagged virus at a MOI of 5. Cells were collected at 16 hpi and fractionated with a Qiagen QProteome Cell Compartment kit. The membrane fraction was immunoprecipitated using an anti-V5 antibody (left panel) or anti-GRP78 antibody (right panel) and the precipitate was analyzed by western blot. (**B**) Vero cells were infected with VEEV-TC83 V5-E2 or VEEV-TC83 at a MOI of 5 and were fixed at 16 hpi. Cells were probed for GRP78 (green), V5 (red), and DAPI-stained. (**C**) Expanded merge image from B. Pearson’s correlation for cell = 0.649025. Scale bar = 10 μm. Representative images from 3 biological replicates are shown.

Having established that inhibition of GRP78 impacts VEEV as well as other alphaviruses, the interaction between GRP78 and the VEEV E2 glycoprotein was further evaluated. Co-immunoprecipitation assays were performed to confirm that GRP78 is an interacting partner of VEEV E2. HEK 293T cells were infected with VEEV TC-83 V5-E2 or the parental VEEV TC-83 at a MOI of 5. After 16 hpi cells were collected and the membrane fraction was isolated using a Qiagen QProteome Cell Compartment kit. The membrane fractions were then immunoprecipitated using an anti-V5 antibody. The precipitate was analyzed by western blot and a band for GRP78 was observed in the cell lysates from cells infected with VEEV TC-83 V5-E2 but not in the control (Figure 4A, left panel). A reverse co-immunoprecipitation assay using anti-GRP78 antibody confirmed the interaction between VEEV E2 protein and GRP78 (Figure 4A, right panel). Fluorescent microscopy was also used to confirm the protein-protein interaction in Vero cells. Vero cells were infected with VEEV TC-83 or VEEV TC-83 V5-E2 at a MOI of 5 and after 16 hpi the cells were fixed, permeabilized, and probed for V5 and GRP78. DAPI staining was used to visualize the nucleus. In the merged image colocalization between GRP78 and V5-E2 can be seen in yellow and, mainly surrounding the nucleus in the ER compartment (Figure 4B and zoomed image in Figure 4C). Collectively, these results confirm the interaction between GRP78 and the VEEV TC-83 E2 glycoprotein seen in the mass spectrometry results.

### 2.6. siRNA Mediated Knockdown of GRP78 Inhibits VEEV TC-83

**Figure 5:**
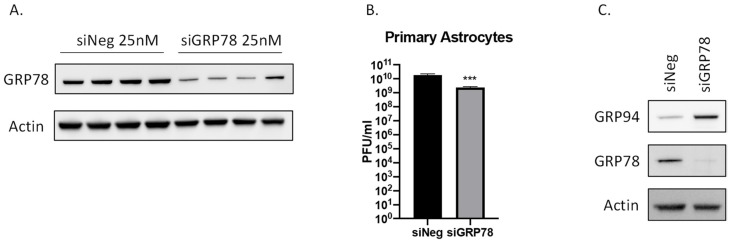
siRNA Mediated Knockdown of GRP78 Inhibits VEEV TC-83. (**A**) Primary human astrocytes were transfected with 25 nM siRNA for 48 hrs and subsequently infected with VEEV TC-83 (MOI of 0.1). At 16 hpi cell lysates were collected and analyzed by western blot for GRP78. (**B**) Supernatants from panel **A** were analyzed by plaque assay. Values are an average of 4 biological replicates ± standard deviation. *** *p* < 0.0005 (**C**) Protein lysates were also analyzed for GRP94 following GRP78 siRNA mediated knockdown and infection as described in **A**.

To further confirm the importance of GRP78 for VEEV TC-83 replication, siRNA against GRP78 was used to knockdown protein expression. Primary human astrocytes were transfected with siRNA against GRP78 or a negative control. After a 48-h transfection period the cells were infected with VEEV TC-83 at a MOI of 0.1. At 16 hpi cell lysates were collected and analyzed by western blot which showed that GRP78 expression was successfully reduced (Figure 5A). The supernatants were collected, and the viral titers determined by plaque assay (Figure 5B). On average the titer was reduced by almost half a log in the GRP78 siRNA treated cell from 7.8 × 10^9^ to 3.35 × 10^9^ PFU/mL. Previous studies have shown that loss of GRP78 results in a corresponding increase in GRP94 protein levels as a way to compensate for a loss of GRP78 [16,17,18]. Indeed, GRP94 protein levels were increased following GRP78 siRNA knockdown (Figure 5C). These results suggest that the ability of GRP94 to compensate for loss of GRP78 may be one factor that contributes to the moderate impact of GRP78 siRNA on VEEV replication as compared to the more potent inhibition observed with HA15 treatment. Nonetheless, these data further confirm that inhibition or loss of GRP78 affects VEEV TC-83 production.

### 2.7. HA15 Treatment Has a Significant Impact on Late Steps of the VEEV Life Cycle

**Figure 6:**
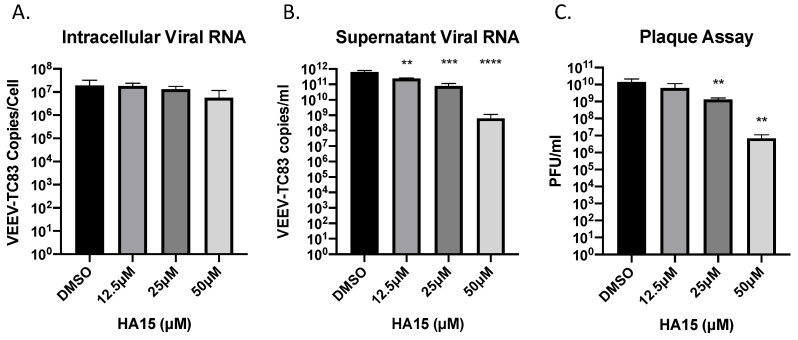
HA15 treatment has a significant impact on late steps of the VEEV life cycle. Vero cells were pretreated with HA15 for one hour and then infected with VEEV TC-83 (MOI of 0.1) for one hour. HA15 was replaced after infection and samples were collected at 16 hpi. (**A**) The intracellular RNA was extracted using a Qiagen RNeasy kit and the amount of viral RNA was determined by RT-qPCR. (**B**) Viral RNA was extracted from the supernatant using a MagMax Viral Nucleic Isolation kit and then the amount of viral RNA was determined by RT-qPCR. (**C**) Supernatants were analyzed by plaque assay to determine the number of infectious particles. Values are an average of 5 biological replicates ± standard deviation. ** *p* < 0.01, *** *p* < 0.0005, **** *p* < 0.0001.

Since HA15 significantly reduced VEEV TC-83 infectious titers, the effects on the virus were further assessed by measuring the levels of viral RNA present intracellularly or extracellularly via RT-qPCR. Vero cells were pre- and post-treated with HA15 and at 16 hpi viral RNA was isolated from the cells or the cell free supernatant. RT-qPCR analysis indicated that intracellular RNA was marginally impacted in cells treated with HA15, signifying that HA15 does not affect viral RNA production (Figure 6A). On the other hand, the amount of viral RNA found in the supernatant was significantly reduced by almost one log from 6.17 × 10^11^ to 7.88 × 10^10^ VEEV-TC83 copies/mL at 25 µM HA15 and almost 3 logs at 50 µM HA15 to 6.45 × 10^8^ copies/mL (Figure 6B). Likewise, a dose dependent reduction in viral titers was observed in these samples (Figure 6C). These results show that HA15 does not significantly affect the production of viral RNA but instead impacts a later step of the viral life cycle, such as viral assembly or viral protein trafficking, resulting in the reduction of released viral RNA and the corresponding decrease in viral titers.

## 3. Discussion

The discovery of new treatments for VEEV and other alphaviruses is necessary due to the current lack of approved therapeutic options to combat these viruses. In this study, a VEEV V5-E2 epitope tagged virus was developed to enable the determination of host pathogen interactions and subsequently potential inhibitors to those interactions. The addition of an epitope tag to VEEV E2 streamlined the process of identifying protein interactions especially when antibodies for immunoprecipitation and subsequent proteomic analysis are not readily available. Using this technique combined with mass spectrometry a total of 785 VEEV E2 interacting proteins were identified, 21 of which appeared in all three biological replicates.

From the list of 21 proteins, GRP78, HSP70, ATP synthase, and Rab-1 were further investigated for their importance in VEEV production through the use of readily available small molecule inhibitors. A total of six inhibitors were tested, Fluvastatin (Rab-1 inhibitor), VER155008 (HSP70 inhibitor), BTB06584 (ATP synthase inhibitor), Enterostatin (ATP synthase inhibitor), Metformin (GRP78 inhibitor), and HA15 (GRP78 inhibitor). Interestingly, only HA15 treatment showed a significant reduction in VEEV TC-83 viral titers. HA15 is a potential cancer therapeutic that directly inhibits GRP78 by reducing the ATPase activity of the chaperone [15]. Metformin is an FDA approved drug for the treatment of diabetes that is an indirect inhibitor of GRP78. Leclerc et al. (2013) show that Metformin significantly decreased GRP78 protein levels through AMP activated protein kinase (AMPK) [19]. In the case of Metformin, the lack of inhibition compared to HA15 may be due to the fact that Metformin is an indirect inhibitor of GRP78 and significant treatment time is needed before GRP78 levels are impacted. The lack of viral inhibition by the other inhibitors suggest that HSP70, ATP synthase, or Rab-1 proteins are not used by VEEV to replicate and/or that there is functional redundancy within the host cell that allows the virus to replicate in the presence of these inhibitors.

GRP78 is a member of the HSP70 family of chaperon proteins that has multiple roles within the cell including protein folding, regulation of the unfolded protein response (UPR), and apoptosis. During ER homeostasis GRP78 is bound to the transmembrane UPR sensor proteins: activation transcription factor 6 (ATF6), protein kinase R (PKR)-like endoplasmic reticulum kinase (PERK), and inositol-requiring enzyme 1 (IRE1). During periods of ER stress GRP78 will dissociated from these proteins and begin its chaperone activity in the ER and the UPR sensor proteins are then activated. Activation of the UPR leads to a decrease in protein synthesis while increasing expression of ER chaperones, including GRP78. After prolonged periods of ER stress, apoptosis is triggered via the PERK pathway [20,21]. VEEV and SINV has been found to induce the activation of the PERK arm of the UPR pathway [22,23]. On the other hand, CHIKV inhibits activation of the PERK pathway by suppressing the phosphorylation of eukaryotic translation initiation factor alpha (eIF2α) [22]. Activation of the UPR during alphavirus infection occurs at least in part due to translation of glycoproteins flooding the ER. Results presented within are consistent with a model in which HA15 treatment inhibits GRP78 chaperone activity leading to the development of misfolded viral glycoproteins in the ER and a reduction of infectious viral titer. However, it is possible that the interaction between GRP78 and E2 is not direct. GRP78 has been found in the cytoplasm [24] and the viral cytoplasmic protein nsP1 was found in the E2 interactome analysis. Therefore, another viral or host protein could be facilitating the interaction between E2 and GRP78.

GRP78 has been shown to be important for other viruses including Dengue virus, Ebola virus, Hepatitis B virus, HIV, human cytomegalovirus (CMV), and Zika virus [25,26,27,28,29,30]. There are multiple steps in the viral life cycles that can be targeted such as viral entry and/or attachment, protein production, and viral release and re-infectivity (reviewed in [31]). During CMV infection, GRP78 is used for cytoplasmic virion assembly and egress [28]. Interestingly, GRP78 was found in the virions of Japanese encephalitis virus (JEV) contributing to viral infectivity [32]. GRP78 associates with MERS-CoV and bat coronavirus HKU9 spike proteins, thereby acting as an attachment factor to facilitate viral entry [33]. These studies in combination with the results presented within indicate that GRP78 inhibition through HA15 treatment has broad-spectrum potential for the treatment of multiple viral infections.

One limitation of the current study is that the in vivo importance of GRP78 to VEEV pathogenesis was not tested. However, there is precedence for successfully targeting GRP78 for other viral infections. Phosphorodiamidate morpholino oligomers directed against GRP78 were able to completely protect mice from lethal Ebola virus infection [30]. As HA15 has been used in mice to test its antineoplastic effect and showed no apparent toxicity [15], future studies will determine the ability of HA15 to prevent VEEV pathogenesis in mouse models.

## 4. Materials and Methods

### 4.1. Cell Culture

Vero cells and HEK 293T cells were grown in Dulbecco’s modified minimum essential medium (DMEM) (Quality Biological, 112-013-101, Gaithersburg, MD, USA) supplemented with 10% heat-inactivated fetal bovine serum (FBS) (VWR, 97068-085, Radnor, PA, USA), 1% penicillin and streptomycin antibiotics (Corning 30-002-CI, Corning, NY, USA), and 1% L- glutamine (Corning, 25-005-CI, Corning, NY, USA). Primary human astrocytes were grown in astrocyte growth medium BulletKit (Lonza, CC-3186, Morristown, NJ, USA). All cells were grown at 37 °C in a humidified environment at 5% CO_2_. Cells were plated at 1.5 × 10^4^ cells in a 96-well plate or 3 × 10^5^ in a 6-well plate unless otherwise stated.

### 4.2. Viruses and Infections

The original plasmid containing the infectious cDNA of VEEV TC-83 was obtained from Ilya Frolov at the University of Alabama Birmingham [34] and used as a template to develop the VEEV TC-83 V5-E2 tagged virus where the V5 tag was inserted at the N-terminus using standard cloning methods. Production of viral stocks was accomplished as previously described [35]. CHIKV (181/25) was obtained from Dr. Naomi Forrester, University of Texas Medical Branch, Galveston [36]. SINV EgAr 339 and VEEV Trinidad Donkey (TrD) was obtained from BEI Resources (NR-15695 and NR-332, respectively). EEEV FL93-939 was obtained from Dr. William Klimstra, University of Pittsburg [37]. For viral infection, plates were seeded and incubated overnight. Cells were infected with a multiplicity of infection (MOI) of 0.1 for drug treatment and siRNA, or at 5.0 for all other infections. Cells were infected for one hour at 37 °C. Inoculum was removed and fresh medium was added. Plates were incubated at 37 °C for specified times and the supernatants were collected. The titer was determined via plaque assay as described in [38].

### 4.3. Western Blot Analysis

Cells were lysed in Blue Lysis Buffer composed of 25 mL 2× Novex Tris-Glycine Sample Loading Buffer SDS (Invitrogen LC2676, Waltham, MA, USA), 20 mL T-PER TissueProtein Extraction Reagent (ThermoFisher, 78510, Waltham, MA, USA), 200 µL 0.5MEDTA pH 8.0, 3 complete Protease Cocktail tablets, 80 µL 0.1M Na3VO4, 400 µL 0.1M NaF, and 1.3 mL 1M dithiothreitol. 25 µL of cell lysate was separated by gel electrophoresis on a NuPAGE 4–12% Bis-Tris gel (Invitrogen, NP0322BOX, Waltham, MA, USA) and transferred to PVDF membrane (ThermoFisher, 88518, Waltham, MA, USA). The membrane was blocked in 5% milk in PBS—0.1% Tween (PBST) solution for 30 min at room temperature. Primary antibody was incubated at room temperature for 1hr or overnight at 4 °C in 5% milk PBST. Antibodies were used as follows: Mouse anti-V5 antibody (BioRad, MCA1360, Hercules, CA, USA) at 1:1000, Rabbit anti-GRP78 (Abcam, ab21685, Cambridge, MA, USA) at 1:1000, Rabbit anti-GRP94 (Abcam, ab3674, Cambridge, MA, USA) at 1:2000, actin HRP (Abcam, ab49900, Cambridge, MA, USA) at 1:30,000, Rabbit anti-Calnexin (Santa Cruz Biotechnology, sc11397) at 1:1000, and Mouse anti-V5 HRP (BioRad, MCA1360P, Hercules, CA, USA) at 1:30,000. The membrane was washed three times for five minutes with 5% Milk PBST. Secondary antibody was prepared in 5% Milk PBST and incubated at room temperature for 1hr. Goat anti-Mouse (Invitrogen, 32430, Waltham, MA, USA) was used at 1:1500 and Goat anti-Rabbit (Invitrogen, 32460, Waltham, MA, USA) was used at 1:3000. Membranes were washed twice for five minutes with PBST and twice for five minutes with PBS. SuperSignal West Femto Maximum Sensitivity Substrate kit (ThermoFisher, 34095, Waltham, MA, USA) was used to image blots on a Chemidoc Imaging System (BioRad, 12003153, Hercules, CA, USA).

### 4.4. Immunoprecipitation

Cells were seeded at 5 × 10^6^ in a T-75 flask and incubated at 37 °C overnight. Cells were infected at a MOI of 5 with either VEEV TC-83 or VEEV TC-83 V5-E2. After the specified time, supernatant was removed and 10mls of PBS was added and cells were scraped off the growth surface. Cells were pelleted at 500 g for 10 min and washed twice with cold PBS by resuspending in 2 mL cold PBS and pelleting at 500 g for 4 min. Qiagen Qproteome Cell Compartment kit (Qiagen, 37502, Germantown, MD, USA) was used to extract the membrane fraction of the cell. Anti-V5 antibody (BioRad, MCA1360, Hercules, CA, USA) was added to the membrane fraction and incubated on rotator overnight at 4 °C. Dynabeads Protein G magnetic beads (Invitrogen, 00765965, Waltham, MA, USA) were used to recover immune complex. 50uL of Dynabeads were added to overnight IP sample and incubated at room temperature for 45 min. Beads were washed twice with Tris sodium EDTA (TNE) buffer with 300 mM NaCl and 0.1% NP-40, once with TNE buffer with 150 mM NaCl and 0.1% NP-40, once in TNE buffer with 50 mM NaCl and 0.1% NP-40, and two final washes with PBS. Fifty uL of Blue Lysis Buffer was added to beads and samples were boiled for 10 min.

### 4.5. Mass Spectrometry

Immunoprecipitated proteins on Dynabeads were mixed with 20 µL of 8 M urea and incubated at 50 °C for 5 min. The mixture was spun at 16,000 g for 2 min and the supernatant was transferred to a clean 0.6 mL tube. The proteins in the supernatant were reduced with 10 mM dithiothreitol, alkylated with 50 mM iodoacetamide, and digested with trypsin at 37 °C for 4 h. The sample was desalted by ZipTip, dried in SpeedVac, then reconstituted with 10 µL of 0.1% formic acid for mass spectrometry (MS) analysis. Liquid chromatography coupled tandem mass spectrometry (LC-MS/MS) experiments were performed on an Orbitrap Fusion (ThermoFisher Scientific, Waltham, MA, USA) equipped with a nanospray EASY-nLC 1200 HPLC system. Peptides were separated using a reversed-phase PepMap RSLC 75 μm i.d. × 15 cm long with 2 μm particle size C18 LC column from ThermoFisher Scientific. The mobile phase consisted of 0.1 % aqueous formic acid (mobile phase A) and 0.1% formic acid in 80% acetonitrile (mobile phase B). After sample injection, the peptides were eluted by using a linear gradient from 5% to 50% B over 60 min and ramping to 100% B for an additional 2 min. The flow rate was set at 300 nL/min. The Orbitrap Fusion was operated in a data-dependent mode in which one full MS scan (60,000 resolving power) from 300 m/z to 1500 m/z was followed by MS/MS scans in which the most abundant molecular ions were dynamically selected by Top Speed, and fragmented by collision-induced dissociation (CID) using a normalized collision energy of 35%. “EASY-Internal Calibration”, “Peptide Monoisotopic Precursor Selection” and “Dynamic Exclusion” (10 s duration), were enabled, as was the charge state dependency so that only peptide precursors with charge states from +2 to +4 were selected and fragmented by CID. Tandem mass spectra were searched against the NCBI human database including the VEEV protein sequences using Proteome Discover v 2.3 from the ThermoFisher Scientific. The SEQUEST node parameters were set to use full tryptic cleavage constraints with dynamic methionine oxidation. Mass tolerance for precursor ions was 2 ppm, and mass tolerance for fragment ions was 0.5 Da. A 1% false discovery rate (FDR) was used as a cut-off value for reporting peptide spectrum matches (PSM) from the database search.

### 4.6. Cell Viability Assays

Cells were plated on a 96-well plate and incubated overnight. Medium was removed and new medium containing inhibitor dilutions was added to the plate. Plates were incubated at 37 °C for 24 h. ATP production was used as a measure of cell viability and detected using Promega CellTiter-Glo (Promega, G7570, Madison, WI, USA).

### 4.7. Inhibitor Treatments

Cells were treated with inhibitor (or solvent control) for one hour at the specified concentrations in the appropriate medium prior to infection (pretreatment). Pretreatment was removed and cells were subsequently infected as described. Fresh medium containing inhibitor (or solvent control) was reapplied. HA15 (MedChemExpress, HY-100437, Monmouth Junction, NJ, USA), VER-155008 (MedChemExpress, HY-10941, Monmouth Junction, NJ, USA), BTB06584 (MedChemExpress, HY-15877, Monmouth Junction, NJ, USA), and Enterostatin (Abbiotec, 350165, Escondido, CA, USA) were dissolved in DMSO to a final concentration of 50mM. Metformin (MedChemExpress, HY-17471A, Monmouth Junction, NJ, USA) and Fluvastatin (MedChemExpress, HY-14664A, Monmouth Junction, NJ, USA) were dissolved in water to a final concentration of 100 mM.

### 4.8. Immunofluorescent Microscopy

After infection cells were fixed in 4% paraformaldehyde for 10 min at room temperature followed by permeabilization with 0.1% Triton X-100 in PBS for 10 min at room temperature. The cells were washed three times with PBS and blocked with 1% bovine serum albumin, 0.3M glycine, and 0.01% Triton X-100 in PBS. Cells were washed three times with PBS and incubated with the primary antibodies in 1% BSA and 0.01% Triton X-100 in PBS overnight at 4 °C. Mouse anti-V5 antibody was used at 1:700 and Goat anti-GRP78 was used at 1:1000. Cell were washed three times with 0.01% Triton X-100 in PBS. Secondary antibodies were prepared in 0.01% Triton X-100 in PBS and incubated at room temperature for one hour. Donkey anti-rabbit Alexa Fluor 488 (Invitrogen, A21206, Waltham, MA, USA) and donkey anti-mouse Alexa Fluor 568 (Invitrogen, A10037, Waltham, MA, USA) were used at 1:500. Cells were washed twice with 0.01% Triton X-100 in PBS followed by one five-minute wash with 0.01% Triton X-100 and DAPI at 1:1000 in PBS. Fluorescence Imaging data was obtained using a Nikon Eclipse Ti2-E microscope. Image acquisition and analysis was performed using Nikon NIS-Elements Imaging Software version 5.20.01 (Nikon, Melville, NY, USA).

### 4.9. siRNA Mediated Knockdown

Human primary astrocytes were plated at 2.5 × 10^5^ cells per well in a 24-well plate and transfected with SMARTpool siRNA targeting GRP78 (Horizon, L-008198-00-0005, Lafayette, CO, USA) at 25nM, negative-control siRNA (Horizon, D-001810-01-05, Lafayette, CO, USA), and DharmaFect 1 (Horizon, T-2001-01, Lafayette, CO, USA) following manufacturer recommendations. After 24 h, transfection medium was replaced with complete medium and cultured for an additional 48 h before infection. After infection supernatants were analyzed via plaque assay and cell lysates were analyzed by western blot analysis as described above.

### 4.10. RNA Isolation and RT-qPCR

Total RNA was extracted from Vero cells after mock infection or infection with VEEV TC-83 using the RNeasy mini kit (Qiagen, Germantown, MD, USA) following manufacturer’s instruction. Viral RNA from the supernatants of the infected cells was extracted using the MagMax-96 Viral RNA isolation kit (ThermoFisher, AM1836, Waltham, MA, USA) following manufacturer’s instructions. Reverse-transcription- quantitative PCR (RT-qPCR) was performed using the StepOnePlus^TM^ Real-Time PCR System (ThermoFisher, 437660, Waltham, MA, USA). Viral RNA was detected using Invitrogen’s RNA UltraSense^TM^ One-Step Quantitative RT-PCR System using Integrated DNA Technologies primer pairs (forward, TCTGACAAGACGTTCCCAATCA, and reverse, AATAACTTCCCTCCGACCACA) and TaqMan probe (50 6-carboxyfluorescein-TGTTGGAAGGGAAG ATAAACGGCTACGC-6-carboxy-N,N,N0,N0-tetramethylrhodamine-30) against nucleotides (nt) 7931 to 8005 of the viral sequence. A standard curve was generated using serial dilutions of VEEV TC-83 RNA at known concentrations. Absolute quantification was performed using StepOne software v2.3 based on the threshold cycle relative to the standard curve.

### 4.11. Statistical Analysis

Statistical analysis was calculated using an unpaired, two-tailed Student’s t-test using GraphPad Prism version 8.3.0 for Mac OS X, GraphPad Software, San Diego, CA, USA, www.graphpad.com (accessed on 23 January 2021).

## Figures and Tables

**Table 1:** VEEV E2 protein interactions identified through LC-MS/MS.

Accession #	Description	% Coverage	# of Peptides	PSMs	# of Unique Peptides
**Protein Folding**					
NP_005338.1	78 kDa glucose-regulated protein precursor [Homo sapiens]	28.13	14	18	13
NP_005337.2	heat shock 70 kDa protein 1B [Homo sapiens]	16.69	9	11	6
NP_005518.3	heat shock 70 kDa protein 1-like [Homo sapiens]	8.74	5	5	2
NP_004273.1	BAG family molecular chaperone regulator 2 [Homo sapiens]	7.58	2	3	2
XP_016875853.1	heat shock protein 105 kDa isoform X6 [Homo sapiens]	1.90	1	1	1
NP_001210.1	calumenin isoform a precursor [Homo sapiens]	4.13	1	1	1
XP_016868148.1	calumenin isoform X1 [Homo sapiens]	7.93	1	1	1
**Metabolism/ATP production**					
NP_001244263.1	ATP synthase subunit alpha, mitochondrial isoform b precursor [Homo sapiens]	3.77	2	2	2
NP_001677.2	ATP synthase subunit beta, mitochondrial precursor [Homo sapiens]	22.87	6	7	6
**Translation**					
NP_000995.1	60S acidic ribosomal protein P2 [Homo sapiens]	33.91	3	5	3
NP_000967.1	60S ribosomal protein L12 [Homo sapiens]	23.64	3	3	3
NP_000959.2	60S ribosomal protein L4 [Homo sapiens]	5.15	2	2	2
NP_003964.3	60S ribosomal protein L14 [Homo sapiens]	5.58	1	1	1
**Cytoskeleton**					
NP_001017992.1	beta-actin-like protein 2 [Homo sapiens]	6.91	2	3	0
**Complement**					
NP_001203.1	complement component 1 Q subcomponent-binding protein, mitochondrial precursor [Homo sapiens]	10.64	1	2	1
**Vesicle Transport**					
NP_004729.1	vesicle-associated membrane protein-associated protein B/C isoform 1 [Homo sapiens]	5.76	2	2	2
NP_004152.1	ras-related protein Rab-1A isoform 1 [Homo sapiens]	8.29	1	1	1
**Ubiquitination**					
NP_001268646.1	polyubiquitin-B precursor [Homo sapiens]	20.96	1	1	1
NP_066289.3	polyubiquitin-C [Homo sapiens]	21.02	1	1	1
NP_001129064.1	ubiquitin-40S ribosomal protein S27a precursor [Homo sapiens]	10.26	1	1	1
NP_001307947.1	ubiquitin-60S ribosomal protein L40 isoform 1 precursor [Homo sapiens]	12.50	1	1	1

The “#” is indicating “Accession number”, “number of peptides”, and “number of unique peptides”.

## Data Availability

Data is contained within the article or supplementary material.

## Supplementary Materials

The following are available online at https://www.mdpi.com/2076-0817/10/3/283/s1, Figure S1: Fluvastatin, VER155008, BTB06584, Enterostatin, and Metformin do not Reduce VEEV-TC83 Infectious Titers, Table S1: VEEV E2 Interactome Proteomic Data.
