# Supplementary material for: Proteomic Discovery of VEEV E2-Host Partner Interactions Identifies GRP78 Inhibitor HA15 as a Potential Therapeutic for Alphavirus Infections
Source: Pathogens. 2021 Mar 2;10(3):283. doi: 10.3390/pathogens10030283 (PMC8000471; doi:10.3390/pathogens10030283)
Supplement: Supplementary file 1 [file pathogens-10-00283-s001.zip › Supplemental Fig 1.docx]

***Supplemental Fig 1. Fluvastatin, VER155008, BTB06584, Enterostatin, and Metformin do not reduce VEEV-TC83 infectious titers.*** (A – E) Cytotoxicity of inhibitors was determine in Vero cells using a CellTiter-Glo Luminescent Cell Viability Assay after 24 hours of treatment with the inhibitors. (F) Effects of Metformin on viral titers. Cells were pretreated for 48hrs and then infected with VEEV TC-83 (MOI of 0.1) for one hour. After infection the inhibitor was reapplied and the supernatant was collected at 16hpi. Viral infectivity was measured by plaque assay. (G – J) Effects on the inhibitors on viral titers in Vero cells. Cells were pretreated for one hour with the inhibitor and infected as described in F.
